# Supplementary material for: A Treat-to-Target Approach in IBD: Contemporary Real-World Perspectives from an International Survey
Source: J Clin Med. 2025 Jan 21;14(3):667. doi: 10.3390/jcm14030667 (PMC11818273; doi:10.3390/jcm14030667)
Supplement: Supplementary file 1 [file jcm-14-00667-s001.zip › jcm-3365047-supplementary.pdf]

Figure S1. Assessment of histology in IBD before starting an advanced therapy in A) Ulcerative colitis and B) Crohn's disease.

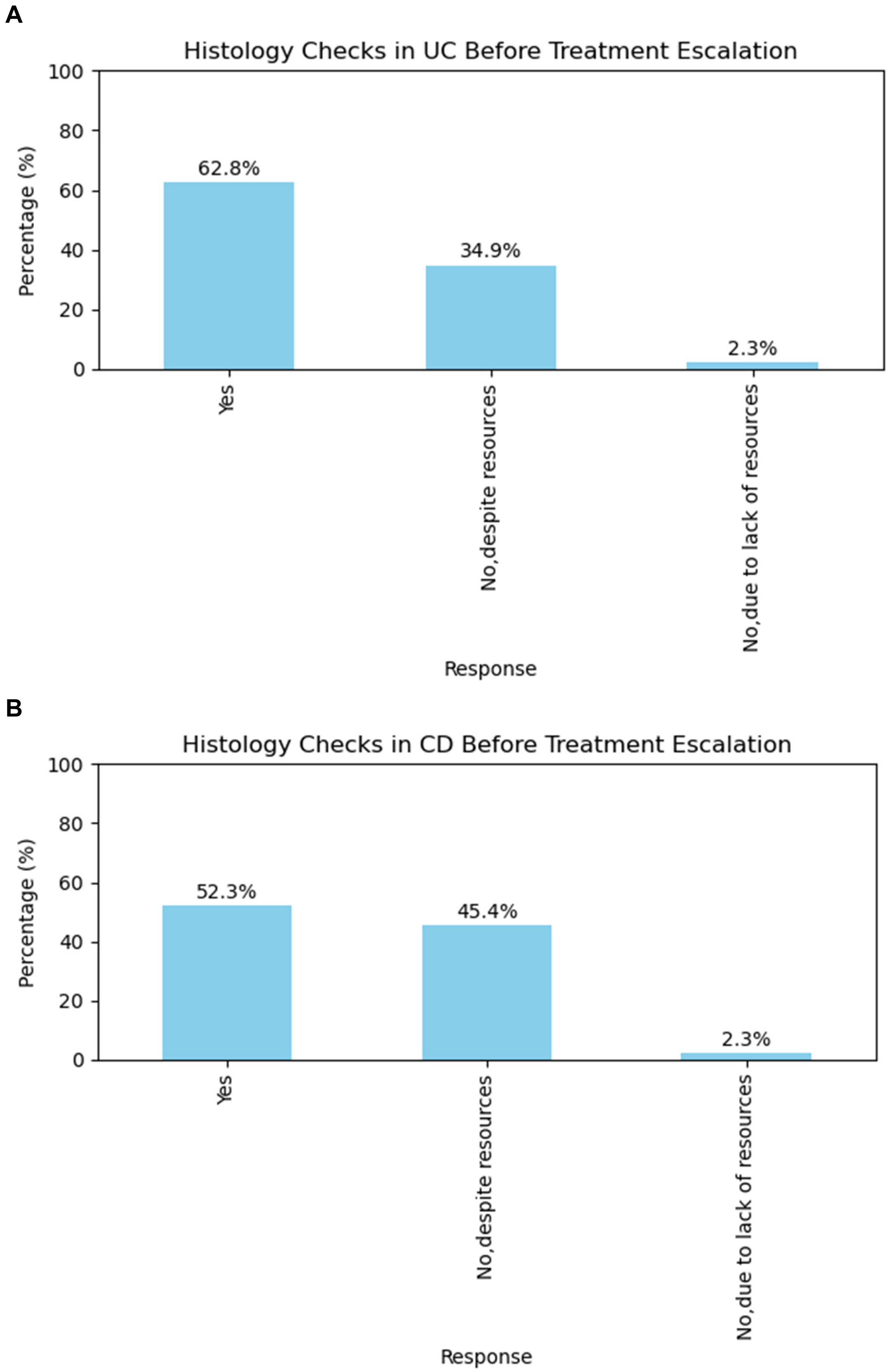

Figure S2. Use of intestinal ultrasound based on country of origin from top five highest respondents in A) Ulcerative colitis and B) Crohn’s disease.

A

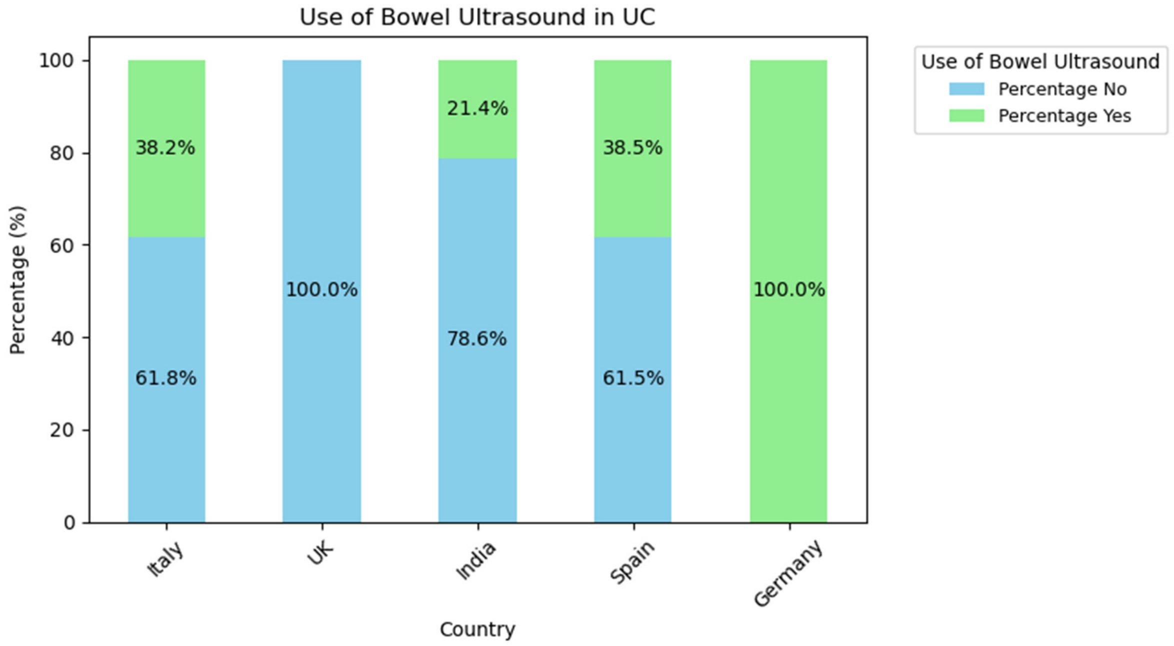

B

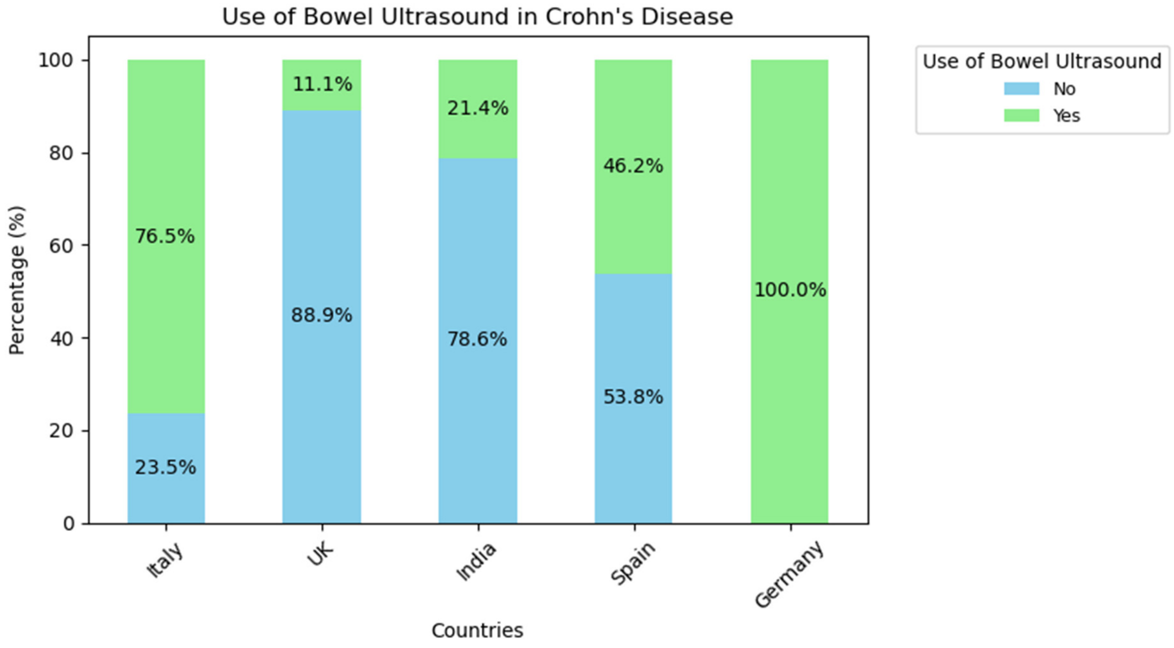

Figure S3. Monitoring of patients with colonoscopy/sigmoidoscopy after starting an advanced therapy in A) Ulcerative colitis and B) Crohn's disease.

A

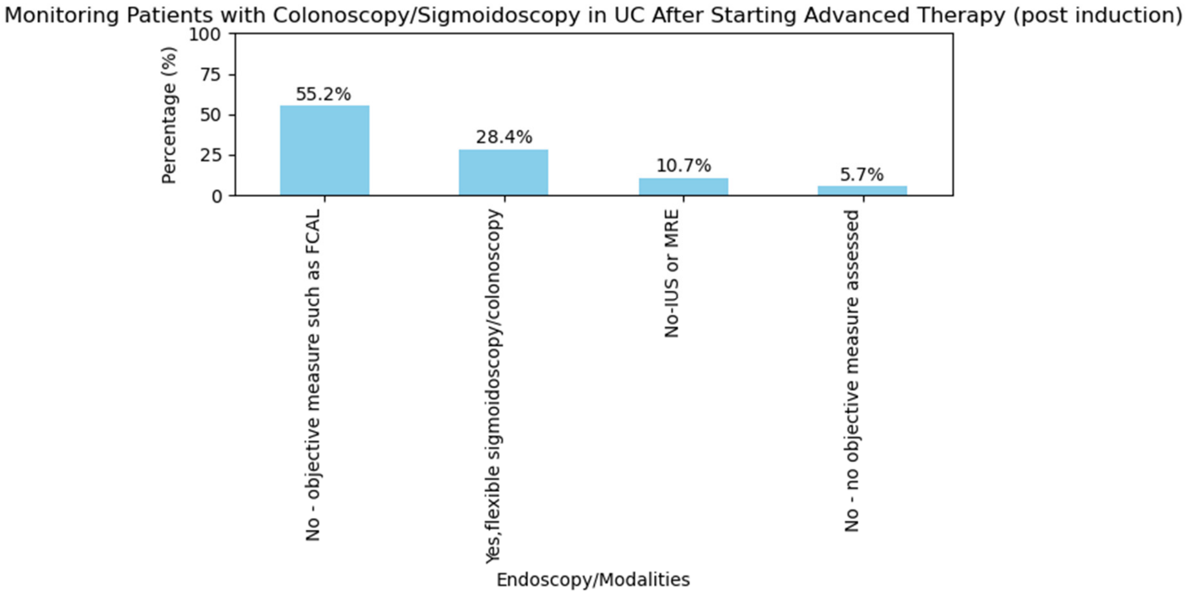

B

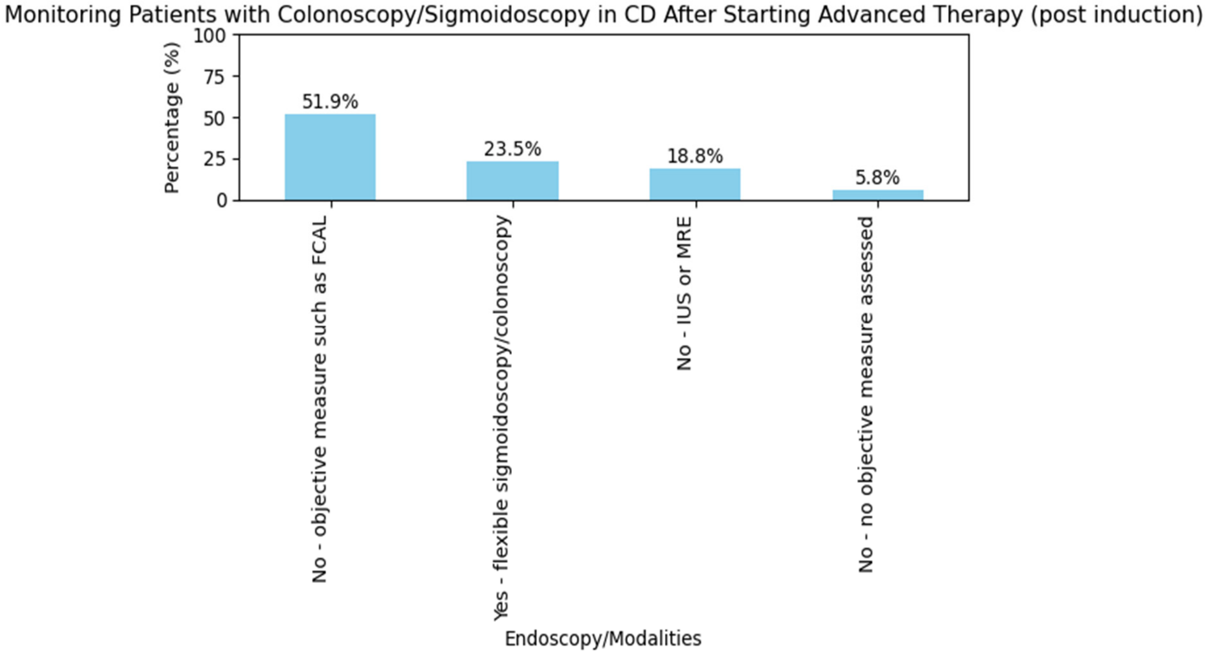

Figure S4. Therapeutic drug monitoring after starting anti-TNF treatment in A) Ulcerative colitis and B) Crohn's disease and C) Top five countries of origin for respondents.

A

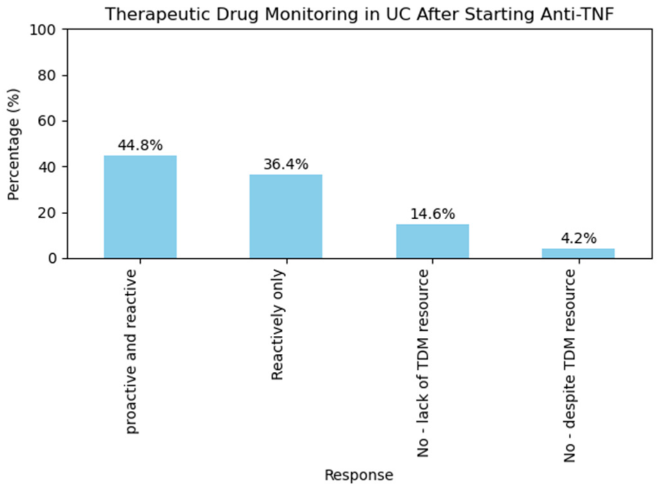

B

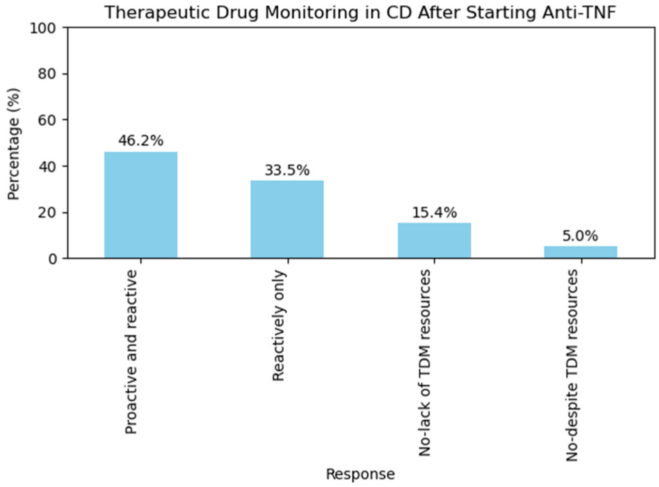

C

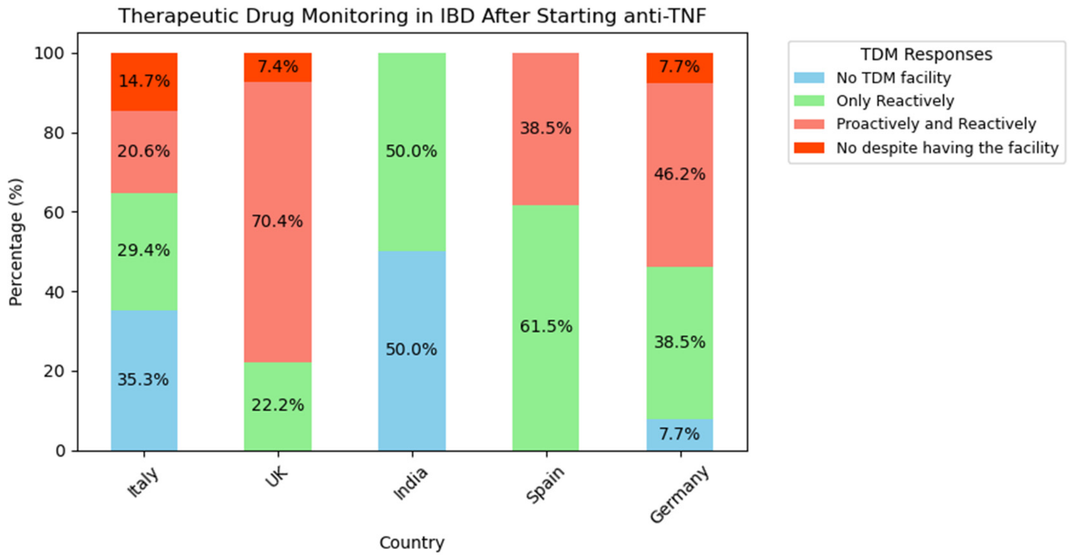

Table S1. Odds ratios for performing intestinal ultrasound based on country of origin for survey respondents in A) Ulcerative colitis and B) Crohn's disease.

A

**Country**	**Odds Ratio**	**P-value**
United Kingdom	0.00	0.0000020
Italy	1.35	0.5109051
Germany	inf	0.0000001
Spain	1.27	0.7570224
India	0.49	0.3728632

B

**Country**	**Odds Ratio**	**P-value**
United Kingdom	0.067	0.000001
Italy	5.46	0.000287
Germany	inf	0.000102
Spain	0.81	0.774567
India	0.22	0.022833

Table S2. Contingency table of χ^2 for use of IUS and country of the respondents in A) Ulcerative Colitis and B) Crohn's disease.

A

Country	IUS Used in UC	IUS Not Used in UC
UK	0	27
Italy	13	21
Germany	13	0
Spain	5	8
India	3	11

B

Country	IUS Used in CD	IUS Not Used in CD
UK	3	24
Italy	26	8
Germany	13	0
Spain	6	7
India	3	11

Table S3. Contingency tables of χ^2 on use of therapeutic drug monitoring in A) Ulcerative colitis and B) Crohn's disease.

A

Country	TDM done proactively and reactively	TDM done reactively only
UK	19	6
Italy	7	10
Germany	6	5
Spain	5	5
India	0	7
**Total**	**37**	**33**

B

Country	TDM done reactively and proactively	TDM done reactively only
UK	20	4
Italy	9	8
Germany	6	5
Spain	8	5
India	0	7
**Total**	**43**	**29**

Table S4. Fisher's exact test results for use of therapeutic drug monitoring in A) Ulcerative colitis and B) Crohn's disease.

A

Country	Odds Ratio	P-value
United Kingdom	4.75	0.0056
Italy	0.53	0.4028
Germany	1.08	1.0000
Spain	0.87	1.0000
India	0.00	0.0035

B

Country	Odds Ratio	P-value
United Kingdom	5.43	0.0048
Italy	0.69	0.5778
Germany	0.77	0.7467
Spain	1.09	1.0000
India	0.00	0.0010

Supplementary Methods
Survey Questions:

Country of Practice:
Current role:

Ulcerative colitis
1. Does your local IBD team routinely perform a flexible sigmoidoscopy/colonoscopy before escalating to an advanced therapy in ulcerative colitis?
a. Yes
b. No

2. Does your local IBD team routinely perform bowel ultrasound before escalating to an advanced therapy in ulcerative colitis?
a. Yes, and hence I perform less endoscopies
b. Yes, but it does not affect my endoscopy uptake
c. No, although we do have the resources for ultrasound
d. No, because we don't have the resources for ultrasound

3. Does your local IBD team routinely check histopathology before escalating to an advanced therapy in ulcerative colitis?
a. Yes
b. No, although we do have the resources for histopathology
c. No, because we don't have the resources for histopathology

4. How does your local IBD team typically monitor treatment response after starting an advanced therapy in ulcerative colitis during induction? (You can select multiple options)
a. Clinical symptoms/ patient reported outcomes (PROs)
b. Faecal calprotectin
d. Serum CRP
c. Flexible sigmoidoscopy/ colonoscopy
e. Bowel ultrasound
f. Histopathology

5. Regardless of clinical response, does your local IBD team routinely perform an interval flexible sigmoidoscopy/ colonoscopy at the end of induction after starting an advanced therapy in ulcerative colitis ?
a. Yes
b. No, but we perform intestinal ultrasound
c. No, but we have another objective assessment like faecal calprotectin
d. No, we do not perform any objective assessment at the end of induction

6. Does your local IBD team use therapeutic drug monitoring (TDM) for patients on anti-TNF medications in ulcerative colitis ?
a. Yes, proactively and reactively
b. Yes, but only reactively
c. No, we don't believe in TDM

d. No, we would like to but we don't have the resources to check TDM

7. For the majority of patients with ulcerative colitis that your local IBD team look after, what is the typical treatment target one-year after treatment initiation?
a. Symptomatic improvement/ patient reported outcomes (PROs)
b. Steroid-free clinical remission
c. Faecal calprotectin below level as per local/ national/ international guidelines

d. Endoscopic remission
e. Mucosal healing (endoscopic and histologic remission)
f. Disease clearance (clinical and endoscopic and histologic remission)

8. For patients who achieve deep remission (clinical remission + endoscopic remission), does your local IBD team consider electively stopping advanced therapy in ulcerative colitis?
a. Never routinely consider elective discontinuation of an advanced therapy
b. Routinely consider but my view is to usually continue treatment
c. Routinely consider and my view is to usually stop treatment

Crohn's disease:
1. Does your local IBD team routinely perform a colonoscopy/ CT enterography /MRI small bowel before escalating to an advanced therapy?
a. Yes, predominantly colonoscopy
b. Yes, predominantly CT enterography
c. Yes, predominantly MR enterography
d. No, we don't perform any endoscopy or CT/MR enterography and mainly treat based on symptoms +/- calprotectin

2. Does your local IBD team routinely perform bowel ultrasound before escalating to an advanced therapy in CD?
a. Yes, and hence we perform less endoscopies/MRE
b. Yes, but it does not affect our endoscopy/MRE uptake
c. No, although we do have the resources for ultrasound
d. No, because we don't have the resources for ultrasound

3. Does your local IBD team routinely check histopathology before escalating to an advanced therapy in CD?
a. Yes
b. No, although we do have the resources for histopathology
c. No, because we don't have the resources for histopathology

4. How does your local IBD monitor the treatment response in Crohn's disease after starting an advanced therapy during induction? (You can select multiple options)
a. Clinical symptoms/ patient reported outcomes (PROs)
b. Faecal calprotectin
c. Serum CRP
d. Colonoscopy
e. Intestinal ultrasound
f. MR enterography
g. Histopathology

5. Regardless of clinical response, does your local IBD team routinely perform a colonoscopy at the end of induction after starting an advanced therapy in Crohn's disease?
a. Yes
b. No, but we perform intestinal ultrasound or MRE routinely
c. No, but we check another objective assessment like faecal calprotectin
d. No, we do not perform any objective assessment at the end of induction

6. Does your local IBD team use therapeutic drug monitoring (TDM) for patients on anti-TNF medications with Crohn's disease?
a. Yes, proactively and reactively
b. Yes, but only reactively
c. No, we don't believe in TDM

d. No, we would like to but we don’t have the resources to check TDM

7. For the majority of patients your local IBD team looks after with Crohn’s disease, what is the typical treatment target at one year?
a. Symptomatic improvement/ patient reported outcomes (PROs)
b. Steroid-free clinical remission
c. Faecal calprotectin below level as per local/ national/ international guidelines
d. Endoscopic remission
e. Mucosal healing (endoscopic and histologic remission)
f. Transmural remission using (CT/MRI/US)

8. For patients who achieve deep remission (clinical remission + endoscopic remission), does your local IBD team consider electively stopping advanced therapy in Crohn’s disease?
a. Never routinely consider elective discontinuation of an advanced therapy
b. Routinely consider but my view is to usually continue treatment
c. Routinely consider and my view is to usually stop treatment
